# Computational chemistry‐assisted design of hydrazine‐based fluorescent molecular rotor for viscosity sensors

**DOI:** 10.1002/smo.20230011

**Published:** 2023-10-12

**Authors:** Miao Dong, Dazhuang Wang, Jinrong Yang, Pingping Sun, Weilu Ding, Jianxin Yang, Jinwu Yan, Weijie Chi

**Affiliations:** ^1^ Collaborative Innovation Center of One Health, Key Laboratory of Green Catalysis and Reaction Engineering of Haikou School of Chemistry and Chemical Engineering Hainan University Haikou China; ^2^ MOE International Joint Research Laboratory on Synthetic Biology and Medicines School of Biology and Biological Engineering South China University of Technology Guangzhou China; ^3^ Beijing Key Laboratory of Ionic Liquids Clean Process CAS Key Laboratory of Green Process and Engineering State Key Laboratory of Multiphase Complex Systems Institute of Process Engineering Chinese Academy of Sciences Beijing China

**Keywords:** computational chemistry, fluorescence probes, fluorescence quenching mechanisms, twisted intramolecular charge transfer

## Abstract

Deep understanding of the fluorescence quenching mechanisms of probes plays a crucial role in developing their practical applications. The fluorescence quenching mechanism of hydrazine‐based fluorescence probes needs to be clarified up to the present. Herein, we designed and synthesized a new hydrazine‐based fluorescence probe (**HA‐Na**) based on the naphthalimide skeleton. We clarified the molecular origin of the non‐fluorescence of this probe with the aid of computational chemistry and spectroscopic analysis. We showed that the significant rotation of the hydrazine group in the excited state potential energy surface, which caused the complete charge separation, was responsible for the fluorescence quenching of the probe in an organic solvent. Once the rotation was prevented in an aggregative state or high‐viscosity solution, the fluorescence of the probe recovered. In other words, the fluorescence quenching mechanism of hydrazine‐based fluorescence probes was attributed to the formation of a twisted intramolecular charge transfer (TICT) state. More importantly, we demonstrated that this fluorescence molecular rotor could be used to monitor the autophagy process in living cells by detecting lysosomal viscosity changes during starvation. Altogether, this work provides an essential theoretical basis for the developing potential hydrazine‐based fluorescence molecular rotors.

## Graphical abstract

Understanding probes' fluorescence quenching mechanisms is crucial in developing their practical applications. Herein, with the aid of computational chemistry, we demonstrated that the formation of twisted intramolecular charge transfer is responsible for the non‐fluorescence of hydrazine‐based fluorescent probes. Moreover, we suggested that a hydrazine‐based fluorescence molecular rotor could be used to monitor the autophagy process in living cells.

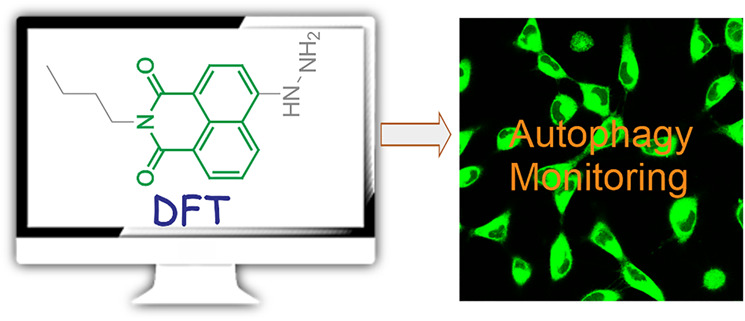

## 1 INTRODUCTION

Fluorescence imaging techniques have become a powerful tool for research in analytical detection, medicine, biology, and pathology.[^1^, ^2^, ^3^, ^4^, ^5^] Fluorescent probes as core units are crucial in increasing imaging quality and detection sensitivity.[^6^, ^7^, ^8^, ^9^, ^10^] The photoluminescence mechanisms of dyes determine their applications.[^11^, ^12^] Yet, the working mechanisms of many dyes remain debatable so far. Deep understanding of these mechanisms is vital for the rational development of functional fluorescent probes.

The hydrazine group has been frequently used to construct “turn‐on” fluorescent probes, in which the hydrazine group acted the roles of recognition and fluorescence quenching groups. However, the quenching mechanism of the hydrazine group to fluorescent probes was unclear. In this regard, Yu et al. designed the first hydrazine‐based fluorescence probe for detecting formaldehyde in 2015.^[13]^ Their results demonstrated that the hydrazine unit plays the role of recognition site and quenching groups in the fluorescence probe. They proposed that the photoinduced electron transfer (PET) process quenched the fluorescence of the probe, which led to a very weak emission. When the hydrazine reacts with formaldehyde and generates methylenehydrazine, the PET process is prevented, and the methylenehydrazine‐based fluorescent dye shows high fluorescence. Lin's group also adopted the hydrazine group to design the hydrazine‐based formaldehyde fluorescence probe (Na‐FA) in 2016.^[14]^ Na‐FA was developed by connecting the hydrazine group to the naphthalimide unit. Following the pioneer report, many hydrazine‐based formaldehyde fluorescent probes were reported based on the naphthalimide skeleton.[^15^, ^16^, ^17^, ^18^, ^19^] Though convincing evidence is lacking, the PET effect has been regarded as the default fluorescence quenching mechanism in these hydrazine‐based fluorescent probes. Besides, abundant hydrazine‐based fluorescent probes based on different fluorophores have also been reported.^[20]^ Suhua^[21]^ and Tang^[22]^ designed the hydrazine‐based fluorescent probes, namely FAP and TFCH, based on the NBD (7‐nitrobenzo‐2‐oxa‐1,3‐diazolyl) and coumarin, respectively. The working mechanism of FAP and TFCH is also considered to be the PET effect, although they did not provide strong experimental or theoretical evidence. However, a different fluorescence quenching mechanism of a hydrazine‐based fluorescence probe was proposed by Song et al. in 2018.^[23]^ They reported a hydrazine‐based fluorescence probe (BHA) based on the BODIPY skeleton. They suggested that the twisted intramolecular charge transfer (TICT) is responsible for the non‐emission of BHA. Even now, the fluorescence quenching mechanism of hydrazine‐based fluorescent probes is still debated.

**SCHEME 1:**
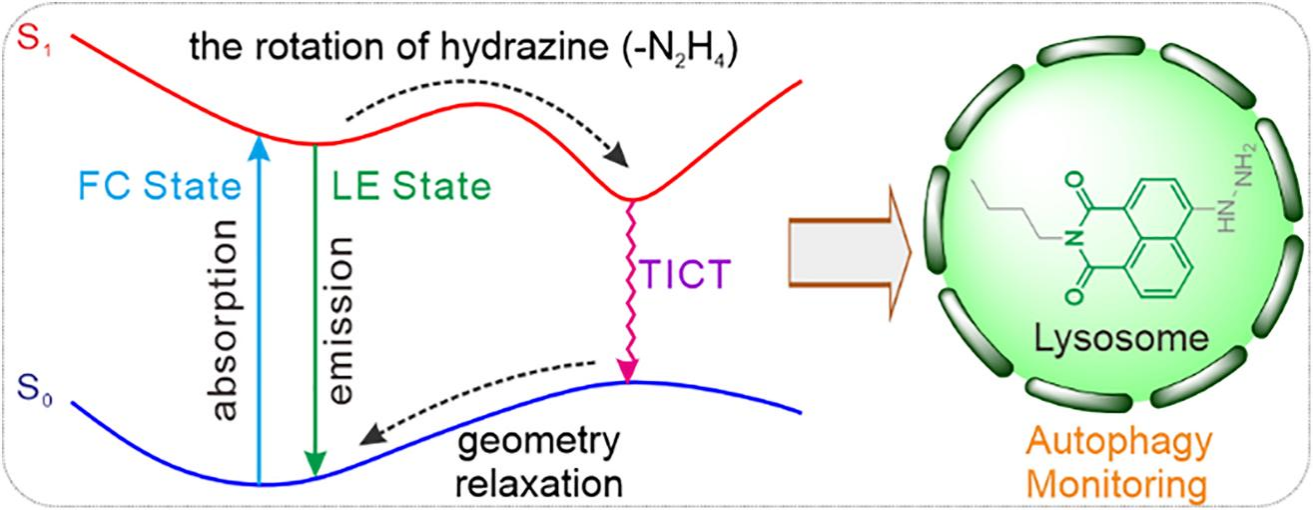
Schematic diagram of twisted intramolecular charge transfer mechanism and monitoring autophagy.

Herein, we focus on the fluorescence quenching mechanism of representative hydrazine‐based fluorescent probes using computational chemistry tools and experimental verification methods. The theoretical results did not support the PET quenching mechanism. Our calculations showed that the rotation of hydrazine groups resulted in the low‐lying excited state geometries for hydrazine‐based fluorescent probes. In these geometries, the hydrazine groups were almost perpendicular to the fluorophores, which led to the complete charge separation of hydrazine‐based fluorescent probes in the excited state along with an oscillator strength of zero. In other words, the rotation of hydrazine groups resulted in the formation of TICT, which is responsible for the fluorescence quenching of hydrazine‐based fluorescent probes (Scheme 1). We designed and synthesized an analog FAP based on a naphthalimide unit, named **HA‐Na**, to verify these calculations. The spectral analysis of **HA‐Na** confirmed the TICT mechanism, which supported these calculations. Finally, the **HA‐Na** has been successfully applied for fluorescence imaging lysosomal viscosity and monitoring autophagy (Scheme 1).

## 2 RESULTS AND DISCUSSIONS

### 2.1 Theoretical verification of PET and TICT mechanisms

Before starting our work, we reviewed the pictures of PET and TICT mechanisms (Figure S1).[^24^, ^25^] Considering the architectural characteristic of investigative molecules, in which the quencher group directly links to the fluorophore by a C‐N single bond, we adopted the picture given by Liu to illustrate the PET process.^[26]^ A theoretical descriptor, the difference in frontier molecular orbital energy (Δ*E*), was put forward to predict PET ON/OFF in Liu's work. Note that the Δ*E* value is equal to *E*_HOMO,fluorophore_ − *E*_HOMO,quencher_ or *E*_LUMO,quencher_ – *E*_LUMO,fluorophore_, whichever is smaller.^[26]^ When Δ*E* < ∼0.6 eV, the PET effect of a fluorophore is activated (PET ON, Figue S1a), leading to a lower fluorescence quantum yield; When Δ*E* > ∼0.6 eV, the PET effect is prevented, resulting in a strong emission (PET OFF, Figure S1a). Hence, the PET ON or OFF can be evaluated by calculating Δ*E*. The theoretical descriptor has been successfully adopted to understand the PET effect of fluorescent dyes.[^27^, ^28^]

Then, we remove our discussion to the TICT mechanism. The TICT mechanism is usually observed in the donor‐acceptor (D‐A) system,^[29]^ and the quasi‐rigid D‐A compounds could undergo approximately 90° intramolecular twisting in the excited state (Figure S1b), which lead to a complete charge transfer excited state.^[30]^ The forbidden transition has a strong fluorescence quenching effect of a fluorophore. When the intramolecular twisting was prevented, the fluorescence recovered.

**FIGURE 1:**
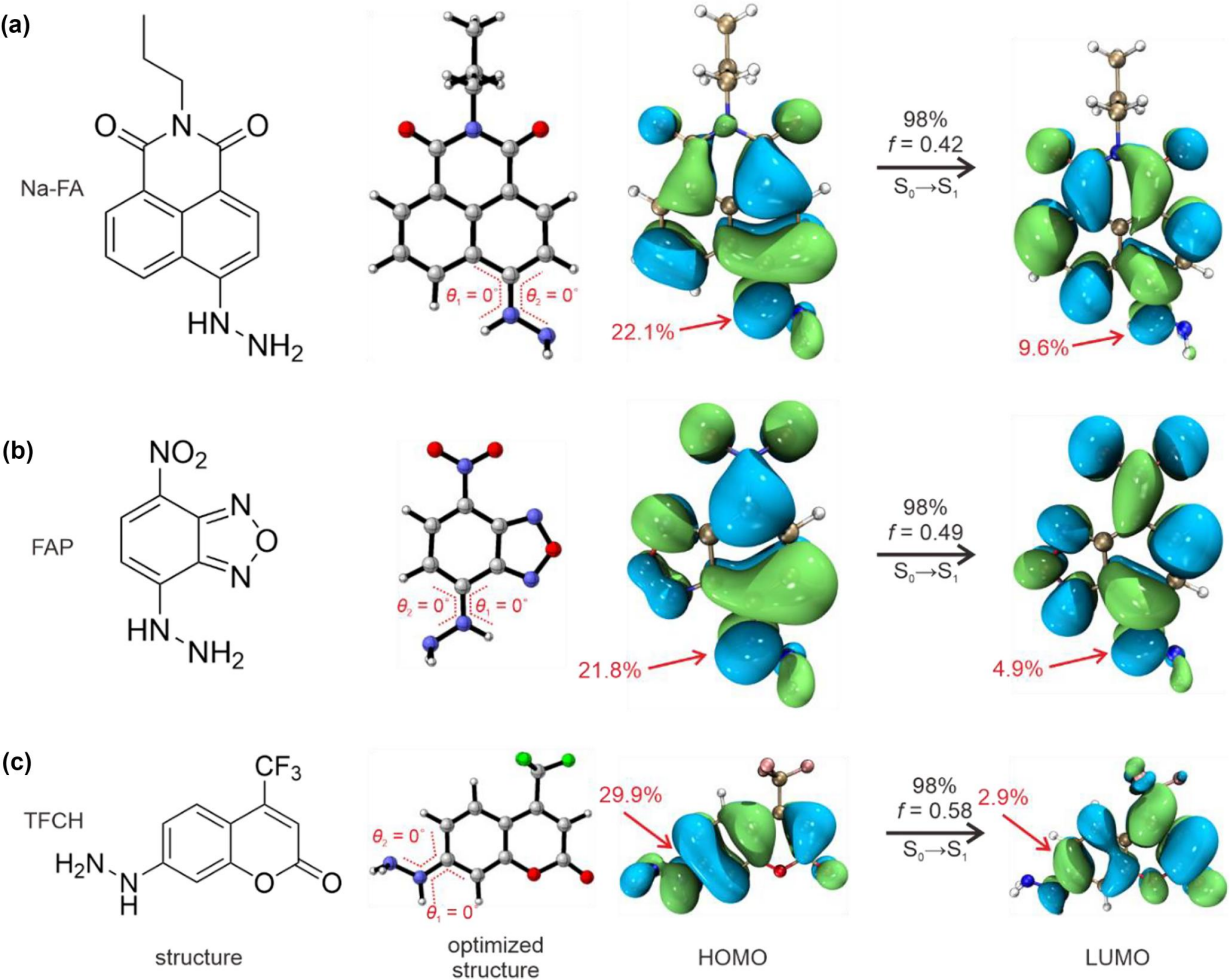
The molecular structures, optimized structures, HOMO, and LUMO of Na‐FA (a), FAP (b), and TFCH (c) at the M062X/TZVP level.

Next, we carefully verified the PEF mechanism of representative hydrazine‐based fluorescent probes, including Na‐FA, FAP, and TFCH, by calculating the distributions of frontier molecular orbit and Δ*E* values. Our calculations demonstrated that the three molecules possessed almost planar configurations in the ground state because of the dihedral angle of 0° between the hydrazine group and fluorophores (Figure 1), which led to a good conjugation effect of these molecules. The strong conjugation caused the broad distributions of the highest occupied molecular orbital (HOMO) and the lowest unoccupied molecular orbital (LUMO). In this regard, the HOMO and LUMO of the three molecules were located on the whole molecules (Figure 1). Moreover, we showed that the contributions of the hydrazine group to HOMO are 22.1%, 21.8%, and 29.9 for Na‐FA, FAP, and TFCH, respectively. However, the contributions to LUMO from the hydrazine group of Na‐FA, FAP, and TFCH reduced to 9.6%, 4.9%, and 2.9%, respectively. The result demonstrated the strong donating‐electron ability of the hydrazine group. Besides, the UV‐vis absorption bands of the three molecules mainly involved the electronic transition from the HOMO to LUMO (∼98%) accompanied by high oscillator strengths, showing the feature of the local excited (LE) state. The direction of electron cloud transfer was from hydrazine groups to fluorophores. Importantly, our results showed that the energy levels of HOMO and LUMO in the hydrazine group (quencher) were far from those of HOMO and LUMO in fluorophores (Figure S2–S4), with larger energy gaps of >1.4 eV (>>0.6 eV). Evidently, the distributions of frontier molecular orbits and energy gaps did not support the PET mechanism. Besides, from an experimental viewpoint, PET dyes' fluorescence intensity was usually sensitive to changes in environmental pH. However, the fluorescence intensity of Na‐FA hardly changed when the pH was raised from 4 to 10 in Lin's work.^[14]^ The PET effect cannot be applied to rationalize the working mechanisms of Na‐FA, FAP, and TFCH.

**FIGURE 2:**
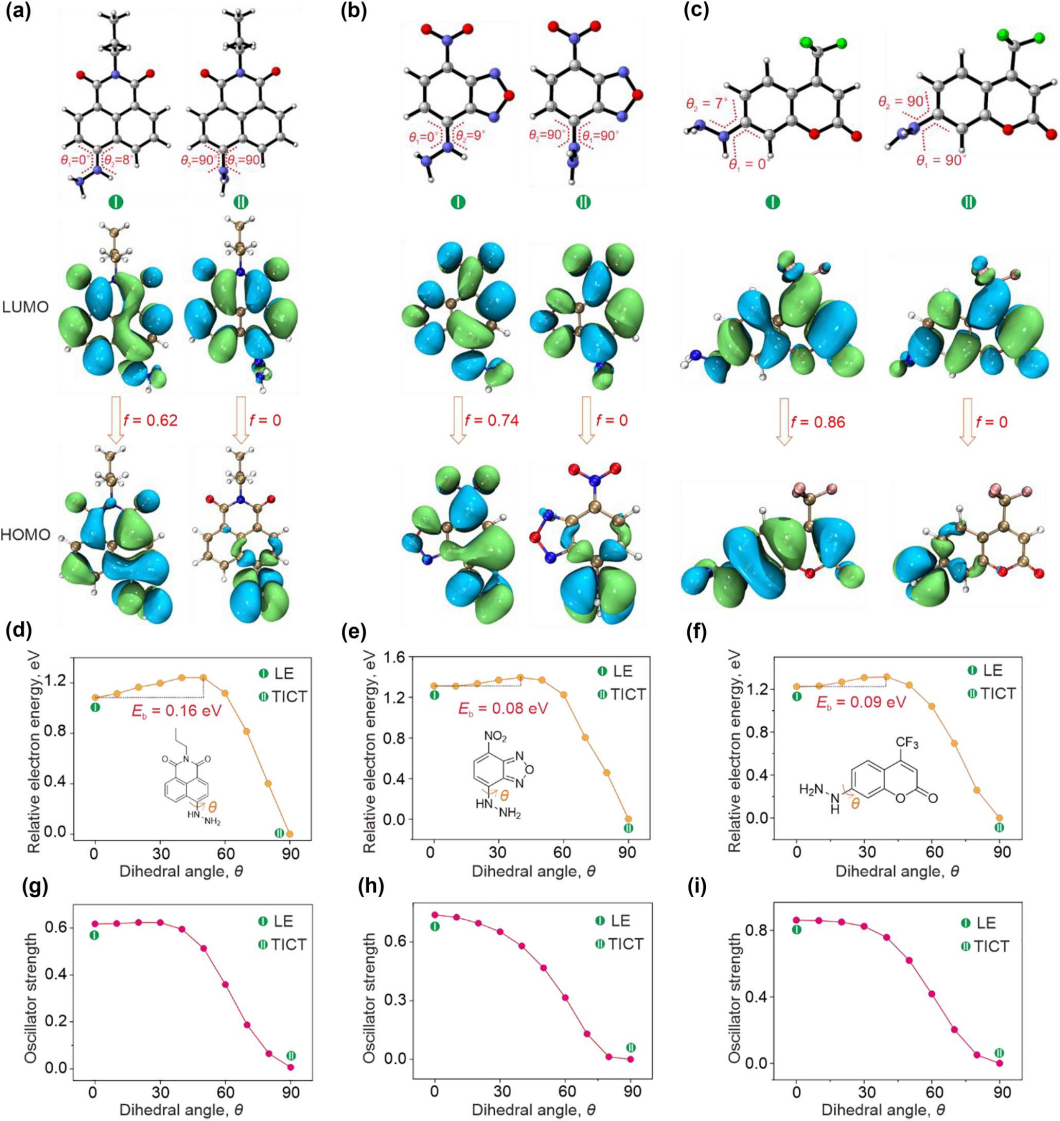
The distributions of HOMO and LUMO in LE and TICT states of Na‐FA (a), FAP (b), and TFCH (c); the excited state PESs of Na‐FA (d), FAP (e), and TFCH (f) as a function of the rotation of the hydrazine group in water solution; and the corresponding oscillator strength of Na‐FA (g), FAP (h), and TFCH (i), all calculations were carried out at M062X/TZVP level.

Then, we confirmed the TICT mechanism of the three molecules. We first optimized the geometries of Na‐FA, FAP, and TFCH in the first excited state (S_1_). We showed that, compared to the geometries of three molecules in the ground state, the *θ*_1_ values are still 0 in the excited state, while the *θ*_2_ values increased to 8°, 9°, and 7° for Na‐FA, FAP, and TFCH, respectively (Figure 2a–c). Although there exists a slight difference between ground and adiabatic excited states in conformations, the feature of electronic transition still belonged to local excited because of the high overlap of HOMO and LUMO (Figure 2a–c). Given the strong electron‐donating strength of the hydrazine group, we speculated that a strong push‐pull effect might cause a structural twisting between hydrazine groups and fluorophores, leading to the formation of the TICT state. Hence, we constructed the potential energy surfaces (PESs) along the rotation (from 0° to 90°) of the hydrazine group in the excited state for Na‐FA, FAP, and TFCH. We showed that indeed the rotation of hydrazine groups, which overcame small energy barrier values (0.08–0.16 eV), resulted in the low‐lying excited state (S_1_)_min_ geometries for three molecules (Figure 2d–f). In the (S_1_)_min_ geometries, the hydrazine groups were almost perpendicular to the fluorophores (Figure 2a–c). Notably, the orthogonal conformation led to the complete charge separation of Na‐FA, FAP, and TFCH in the excited state along with an oscillator strength of 0 (Figure 2g–i). In other words, the rotation of hydrazine groups resulted in the formation of TICT.

To confirm the TICT mechanism, we also calculated the excited‐state PESs of methylenehydrazine‐based fluorescent dyes (Na‐FA‐P, FAP‐P, and TFCH‐P in Figure S5), which were obtained via the reaction of hydrazine‐based fluorescent probes (Na‐FA, FAP, and TFCH) with formaldehyde. Compared with Na‐FA, FAP, and TFCH, the experimental results demonstrated that Na‐FA‐P, FAP‐P, and TFCH‐P showed high brightness in organic solvents.[^14^, ^21^, ^22^] Our calculations suggested that similar to the excited‐state PESs of Na‐FA, FAP, and TFCH, the rotation of the methylenehydrazine group also led to the formation of a non‐emissive TICT state in the excited state (Figure S6–S8). However, it is worth noting that the energy barrier values of entering the TICT state in Na‐FA‐P, FAP‐P, and TFCH‐P (0.18–0.38 eV) were significantly higher than those of Na‐FA, FAP, and TFCH (Figure S9). In other words, only a tiny portion of Na‐FA‐P, FAP‐P, and TFCH‐P may drop the non‐emissive TICT state, and most of these dyes still belonged to the local excited and showed strong fluorescence. In short, these theoretical calculations support the TICT mechanism of hydrazine‐based fluorescent probes.

### 2.2 Spectroscopy verification of TICT mechanism

**FIGURE 3:**
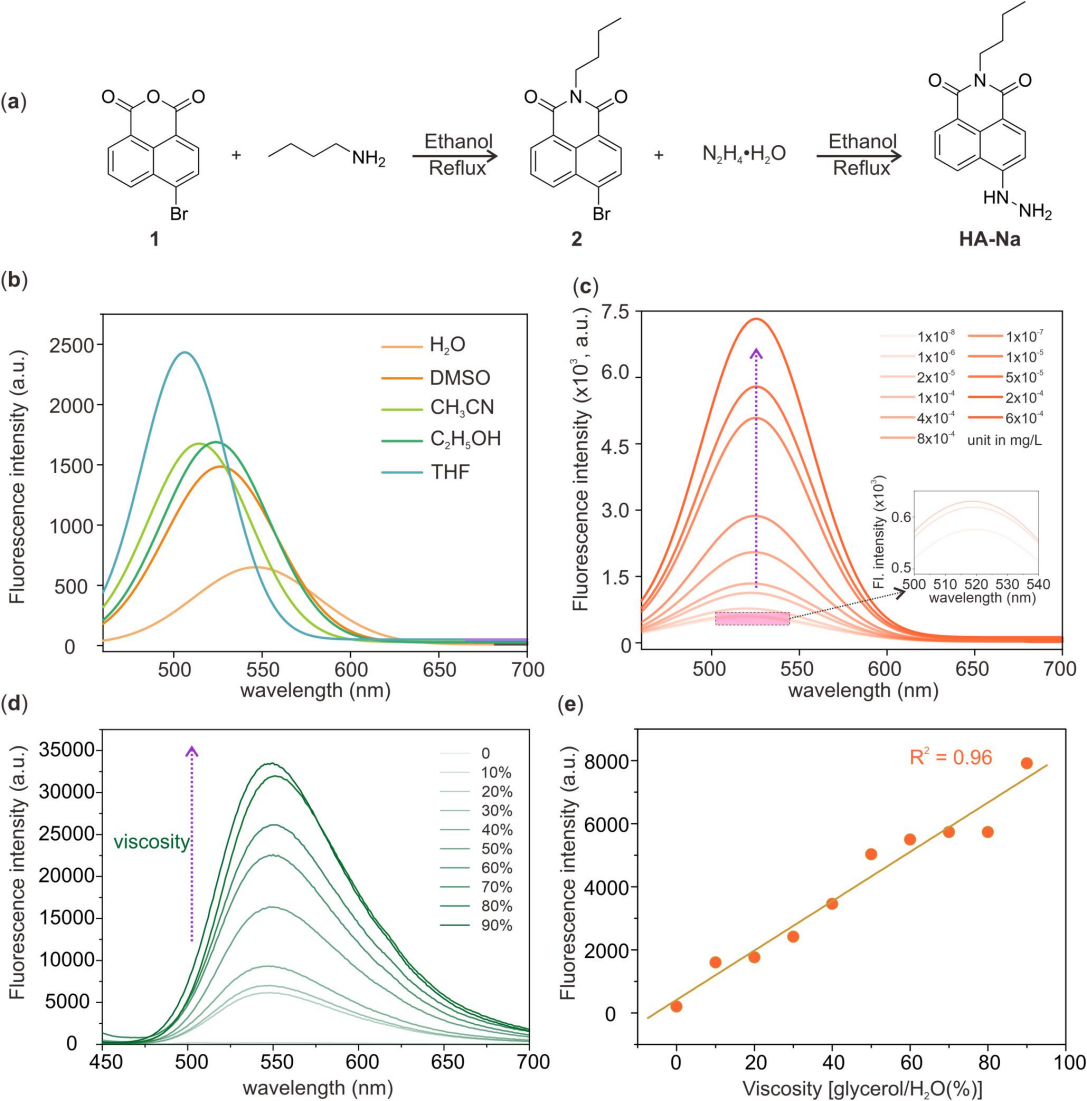
(a) Synthetic process of **HA‐Na**; (b) fluorescence spectra of **HA‐Na** in water, DMSO, acetonitrile, ethanol, and tetrahydrofuran; (c) concentration‐dependent fluorescence spectra of **HA‐Na**; (d) viscosity‐dependent fluorescence spectra of **HA‐Na** in a mixture of water and glycerol; (e) the relationship of fluorescence intensity and viscosity.

In order to verify our calculations, we synthesized and characterized a new compound, namely **HA‐Na** (synthetic method shown in Figure 3a, and experimental section and Figure S10–S15 in *SI*). First, the absorption and emission spectra of **HA‐Na** were recorded (Figure S16 and Figure 3b). The UV‐vis absorption peaks were between 419 and 444 nm in various solvents. The polarity of the solution has a significant effect on the fluorescence wavelengths of **HA‐Na**. Strong polarity leads to a long fluorescence wavelength, showing a significant intramolecular charger transfer (ICT) effect of **HA‐Na**. It is also found that **HA‐Na** possesses low fluorescence intensity (<2500) in the absolute solvent. To verify the origin of the weak fluorescence of **HA‐Na**, we investigated the effects of concentration and viscosity on the fluorescence emission. As the concentration increases, the emission intensity of **HA‐NA** experiences a considerable increase from 1 × 10^−8^ to 6 × 10^−4^ mg/L (Figure 3c). Besides, with the gradual viscosity increase from water to glycerol, the fluorescence shows a distinct increase with the volume fractions of glycerol from 0% to 100% (Figure 3d). Moreover, there is an excellent linear relationship (*R* ^2^ = 0.96) between the fluorescence intensity and viscosity (Figure 3e). The results of the viscosity and concentration responses confirm the AIE effect of **HA‐NA**. Moreover, the change of fluorescence intensities in different viscosities and concentrations also demonstrate the origin of weak fluorescence emission of **HA‐NA** in organic solution. In this regard, the rotation of the hydrazine group leads to the formation of TICT, which reduces the fluorescence intensity. In high concentrations and large viscosity, the rotation of the hydrazine group is prevented, and the fluorescence recovers. These experimental results are in good agreement with the theoretical calculations.

### 2.3 Subcellular imaging

**FIGURE 4:**
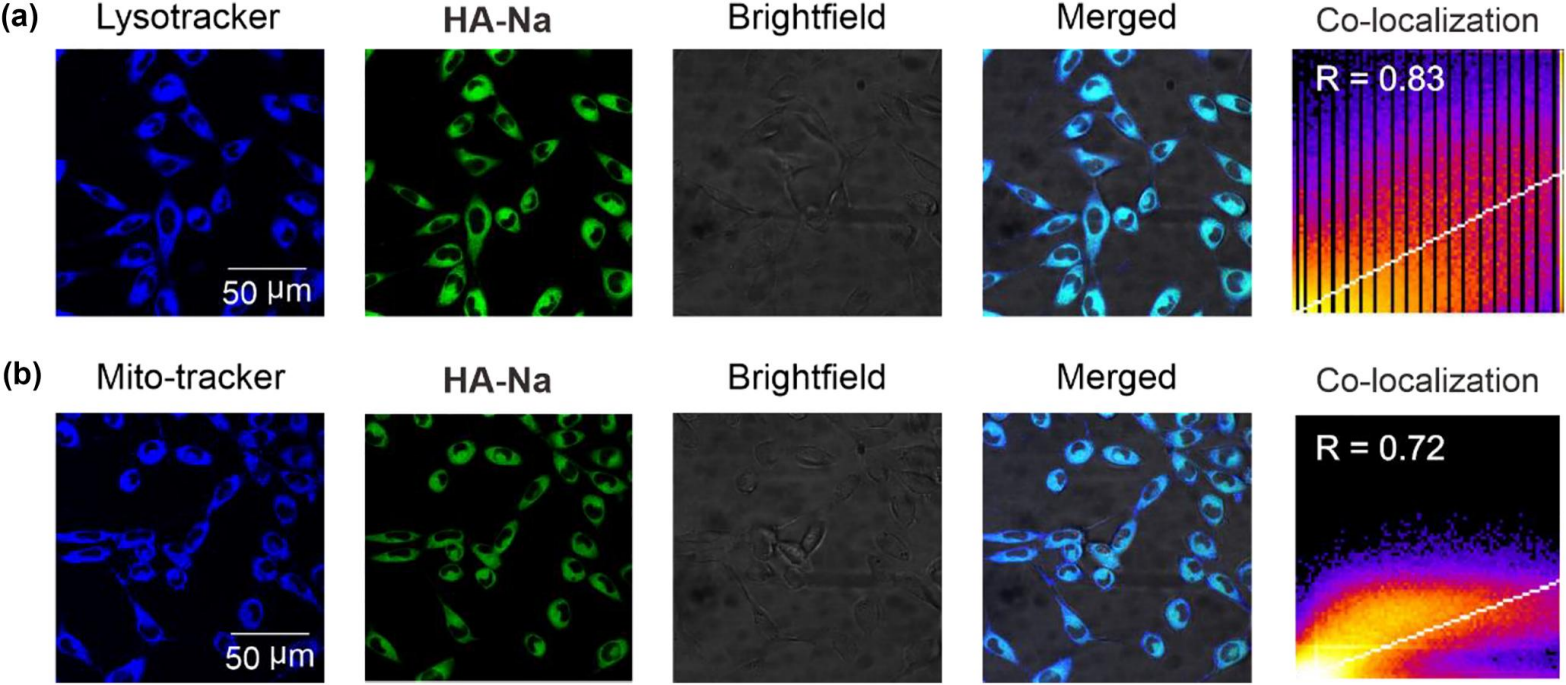
Fluorescence images of Hela cells co‐incubated with **HA‐Na** (1 μM) and (a) Lysotracker blue (10 μM) or (b) Mito‐tracker blue (10 μM) for 1 h at 37°C, respectively, for Lysotracker blue and Mito‐tracker blue, λ_ex_ = 405 nm, λ_em_ = 425−480 nm; for **HA‐Na**, λ_ex_ = 488 nm, λ_em_ = 500−600 nm.

Theoretical and experimental results demonstrate the role of the fluorescence rotor of the hydrazine group in **HA‐Na**. Next, bioimaging experiments are performed to investigate the potential applications of **HA‐Na** in organelle images. We first evaluated the toxicity of **HA‐Na** in cells. The cytotoxicity of **HA‐Na** on Hela cells was determined using the commonly used MTT assay by incubation of **HA‐Na** ranging from 0 to 50 μM for 24 h, and then the data are shown in Figure S17, which demonstrated that the probe had no cytotoxicity on these living cells even in high concentrations, confirming their high potential for bioimaging. The **HA‐Na** and two commercially available trackers, including a lysotracker (lysosome probe) and mito‐tracker (mitochondrion probe), were added to the human breast cancer cell (Hela cell, Figure 4). After incubating for 1 h, we show that the **HA‐Na** successfully enters the cells. The emissions in the range of 500−600 nm (green channel) with excitation at 488 nm were collected. Meanwhile, the lysotracker and mito‐tracker emissions were also recorded in the range of 425−500 nm (blue channel) with excitation at 405 nm. We observed the green fluorescence of **HA‐Na** and blue fluorescence of Lysotracker with an excellent overlap (Pearson's coefficient = 0.83, Figure 4a). In contrast, **HA‐Na** did not significantly stain mitochondrion with a Pearson's coefficient of 0.72 (Figure 4b). The result demonstrates that the **HA‐Na** is highly sensitive to the lysosome. The significant Pearson's coefficient may derive from the low pH of lysosomes (4.5–5.5), and the acidic environment is attractive to certain dyes with amine groups. The ‐NH_2_ group was included in the HA‐Na dye; when **HA‐Na** enters the cell and is transported inside the lysosome, it interacts with the acidic environment inside the lysosome. This interaction may include electrostatic interaction and hydrogen bond interactions, allowing the dyes to aggregate and accumulate within the lysosome.

**FIGURE 5:**
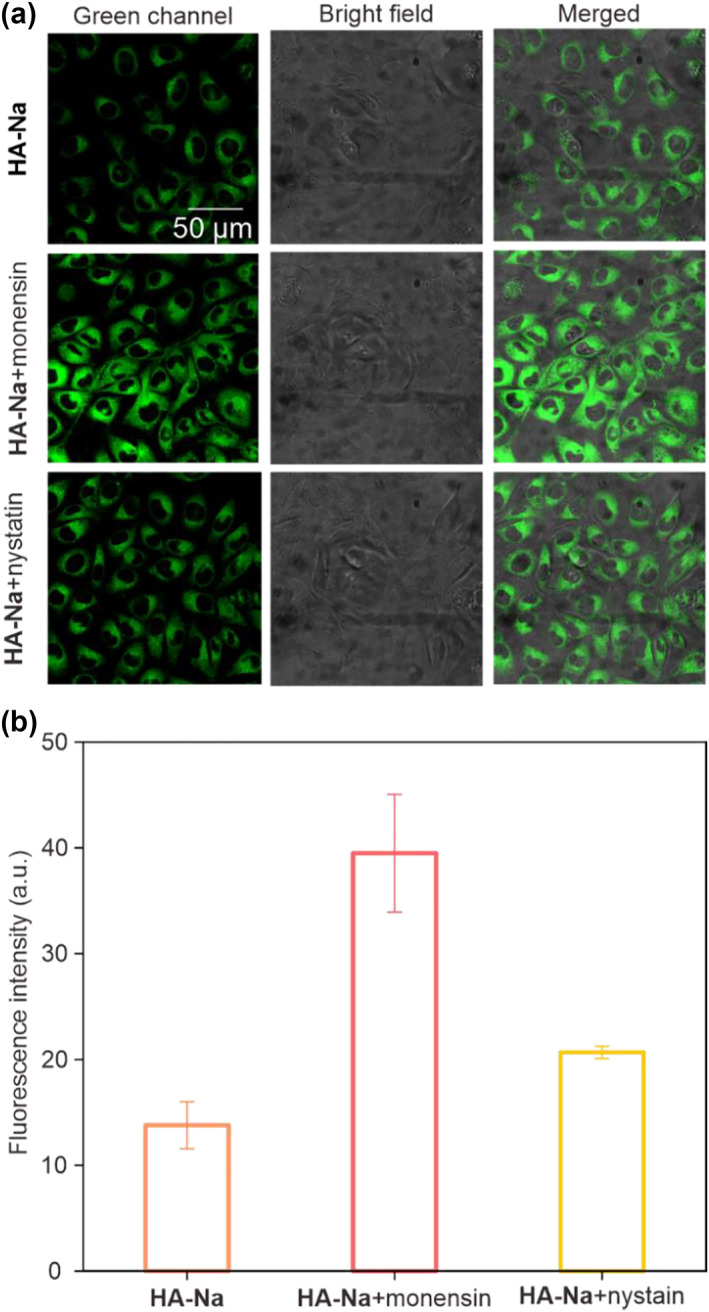
(a) Confocal fluorescence images of **HA‐Na** (10 μM) in Hela cells, which were pre‐treated with normal, monensin, and nystatin (20 μM) for 50 min and then treated with **HA‐Na** (10 μM) for another 1 h (b) the relative fluorescence intensity of **HA‐Na** pretreated with normal, monensin, and nystatin (λ_ex_ = 488 nm, λ_em_ = 500−600 nm).

To further verify the ability to monitor the function of viscosity in biological systems, **HA‐Na** was incubated in the Hela cells under three different conditions, including a normal situation for 1 h, adding monensin for 50 min and adding nystatin for 50 min, for a variation of fluorescence intensity in living cells (Figure 5). Previous reports have shown that monensin and nystatin, as well‐known ionophores, can cause a viscous increase in cells.^[31]^ Our results demonstrated that **HA‐Na** shows weak fluorescence emission in the normal situation (low viscous cells). However, we observed enhanced fluorescence emissions at the 500−600 nm channel after the cells were preincubated with monensin and nystatin (Figure 5a). Especially under monensin conditions, the fluorescence intensity was nearly three times higher than in normal conditions (Figure 5b). The strong emission was mainly derived from the increase in the viscosity of cells. Large viscosity led to a high rotational barrier of the hydrazine group. As a result, the TICT state is thermodynamically inaccessible, thus resulting in strong fluorescence enhancements. Besides, we also excluded the effect of formaldehyde content on the enhancement of fluorescence in **HA‐NA**. We adopted two sensitive fluorescent probes (FFP585 and FFP706) to detect formaldehyde in cells.^[32]^ There was little change in the fluorescence intensity of FFP585 and FFP706 in three conditions, including a normal situation, adding monensin for 40 min, and adding nystatin for 40 min (Figure S18–S19).

### 2.4 Monitoring lysosomal viscosity changes during autophagy

Autophagy is an essential degradation process of cytoplasmic dysfunctional proteins and organelles in eukaryotic evolution.^[33]^ Normal autophagy is connected with many important cellular physiological activities, such as regulating cell survival and death.^[34]^ However, the abnormal autophagy of cells causes many diseases, including cancer, neurological disorders, and aging.^[35]^ Hence, monitoring the autophagy process is significant for bioscience and medicine. During cellular autophagy, the microenvironment of the cell will change because of differences between the autophagosomes and lysosomes,^[36]^ where the cellular viscosity dramatically increases. Considering that **HA‐Na** has a sensitive response to environmental viscosity, we speculated that the probe has potential applications in monitoring the process of autophagy. To verify this conjecture, we used **HA‐Na** to monitor the starvation‐induced autophagy process by monitoring the lysosomal viscosity changes in this process. The Hela cells were incubated with **HA‐Na** (10 μM) at 37°C for 30 min and then cultured in Hank's balanced salt solution (HBSS), normal medium, and HBSS with the addition of 3‐methyladenine (3‐MA) (an autophagy inhibitor) to give the starvation conditions, rich‐nutrient conditions, and autophagy‐inhibited conditions for 90 min, respectively.

**FIGURE 6:**
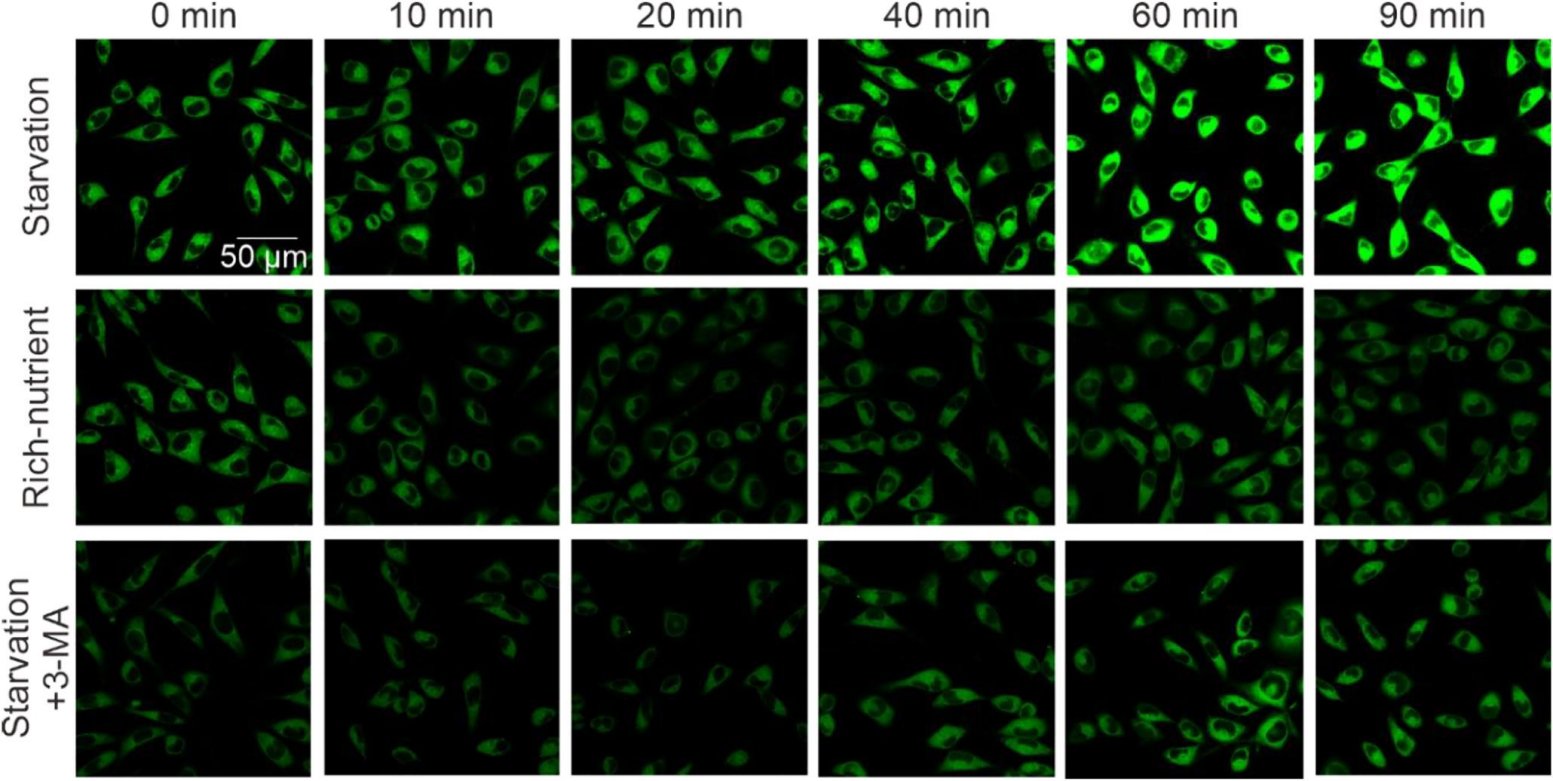
Real‐time confocal fluorescence imaging of **HA‐Na** (10 μM) in Hela cells cultured under starvation conditions (HBSS), nutrient‐rich conditions (normal medium), and autophagy‐inhibited conditions (HBSS with 100 μM 3‐MA) at 0 min, 10 min, 20 min, 40 min, 60 min, and 90 min, respectively.

**FIGURE 7:**
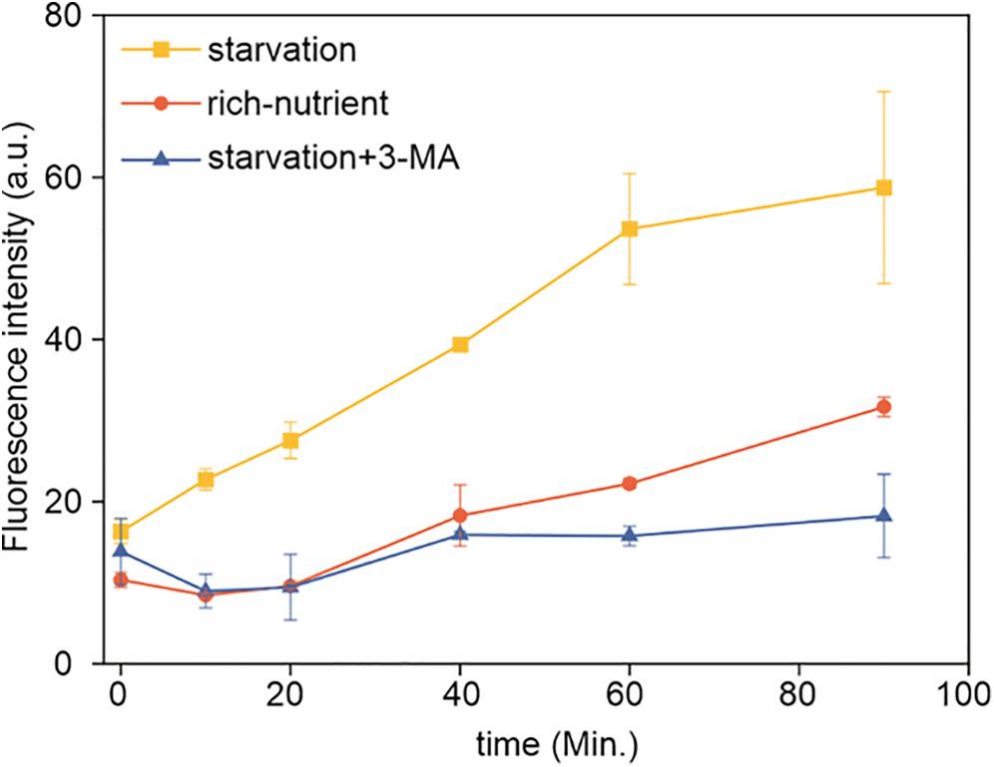
Relative fluorescence intensity of **HA‐Na** (10 μM) at different time points under different conditions (λ_ex_ = 488 nm, λ_em_ = 500−600 nm).

Our results demonstrated that the fluorescence brightness is higher and higher as the time goes from 0 to 90 min in the starvation condition (Figure 6). The strong emission was attributable to the enhanced viscosity induced by starvation in the autophagy process.[^37^, ^38^] In comparison, in the rich nutrient condition, there was only a slight change in the brightness of the fluorescence due to absent autophagy (Figure 7). Besides, when an inhibitor, 3‐methyladenine (3‐MA), was added to the starvation system, it acted to prevent autophagy. The fluorescence brightness was not increased when the time was from 0 to 90 min (Figure 7). These results strongly suggested that **HA‐Na** had great potential in the real‐time monitoring of the autophagy process by tracing the viscosity changes.

## 3 CONCLUSIONS

In conclusion, we designed and synthesized new hydrazine‐based fluorescent probes (**HA‐Na**) based on the naphthalimide skeleton. With the help of density functional theory calculations and spectroscopic analysis, we revealed that the formation of the TICT state quenched the fluorescence of hydrazine‐based fluorescent probes in the organic solvent. However, the rotational barrier of the hydrazine group increased in the aggregative state and high viscosity solution, preventing access to the TICT state, and the **HA‐Na** showed strong emission. Finally, the **HA‐Na** was successfully applied to monitor the autophagy process in living cells by detecting lysosomal viscosity changes during the starvation condition. This work provides some critical information for the development of multifunctional fluorescent molecular rotors. It also showed that experimental verification combined with theoretical calculations is an important research strategy to reveal the photoluminescence mechanisms of dyes and design efficient fluorescent probes.

## CONFLICT OF INTEREST STATEMENT

The authors declare that they have no known competing financial interests or personal relationships that could have appeared to influence the work reported in this paper.

## ETHICS STATEMENT

All animal operations were in conformity to Guidelines for Care and Use of Laboratory Animals of South China University of Technology (SCUT) and approved by animal ethics of SCUT.

## Supporting information

Supporting Information S1

## Data Availability

The data that support the findings of this study are available in the supplementary material of this article.
