# Supplementary material for: Computational chemistry‐assisted design of hydrazine‐based fluorescent molecular rotor for viscosity sensors
Source: Smart Mol. 2023 Oct 12;1(3):e20230011. doi: 10.1002/smo.20230011 (PMC12118249; doi:10.1002/smo.20230011)
Supplement: Supplementary file 1 — Supporting Information S1 [file SMO2-1-e20230011-s001.docx]

***Supporting Information***

Computational Chemistry-assisted Design of Hydrazine-based Fluorescent Molecular Rotor for Viscosity Sensors

Miao Dong,^#a^ Dazhuang Wang,^#a^ Jinrong Yang,^#b^ Pingping Sun,^a^ Weilu Ding,^c^ Jianxin Yang,*^a^ Jinwu Yan *^b^ and Weijie Chi *^a^

^a^ Collaborative Innovation Center of One Health, School of Chemistry and Chemical Engineering, Hainan University, Haikou 570228, China

^b^ MOE International Joint Research Laboratory on Synthetic Biology and Medicines, School of Biology and Biological Engineering, South China University of Technology, Guangzhou 510006, China

^c^ Beijing Key Laboratory of Ionic Liquids Clean Process, CAS Key Laboratory of Green Process and Engineering, State Key Laboratory of Multiphase Complex Systems, Institute of Process Engineering, Chinese Academy of Sciences, Beijing 100190, China

^#^ These authors contributed equally to this work.

*Corresponding author E-mail: [weijie_chi@hainanu.edu.cn](mailto:weijie_chi@hainanu.edu.cn) (W. Chi), [yangjxmail@hainanu.edu.cn](mailto:yangjxmail@hainanu.edu.cn) (J. Yang), yjw@scut.edu.cn (J. Yan)

**1. Experimental section**

1.1. Materials and reagents

4-bromo-1,8-naphthalic anhydride, butylamine, hydrazine hydrate, ethanol, Phosphate Buffer Saline (PBS) and Hank’s balanced salt solution (HBSS) were purchased from Aladdin (Shanghai, China). All materials were used without further purification.

1.2. Synthesis of N-butyl-4-bromo-naphthalimide

4-bromo-1,8-naphthalic anhydride 5.60 g (0.20 mol) and butylamine 1.825 g (0.025 mol) were added to 60 mL of ethanol, and the reaction mixture was continuously stirred and heated to reflux. After the reaction mixture was dissolved, the heating was closed. Numerous solids were precipitated when the reaction solution was cooled to room temperature. Filtered, washed, and pale yellow solids with a yield of 90% were obtained after drying.

^1^H NMR (400MHz, DMSO-d6) δ 8.56 (m, 2H), 8.34 (d, *J* = 7.8 Hz, 1H), 8.23 (d, *J* =7.8 Hz, 1H), 8.00 (t, *J* = 7.8 Hz, 1H), 4.03 (t, *J* = 7.4 Hz, 2H), 1.62 (m, 2H), 1.36 (m, 2H), 0.93 (t, *J* = 7.4 Hz, 3H). ^13^C NMR (100 MHz, DMSO-d6) δ 163.2, 163.1, 132.9, 131.9, 131.7, 131.3, 130.1, 129.6, 129.0, 128.6, 123.1, 122.2, 40.6, 30.1, 20.4, 14.2. HR-MS calculated for C16H14BrNO2 [M+H]^+^ m/z 332.0286, found 332.0285.

1.3. Synthesis of N-butyl-4-hydrazino-naphthalimide

A mixture of N-butyl-4-bromo-naphthalimide 1.30 g (0.0041 mol) and 10 mL 80% hydrazine hydrate in 20 mL ethanol was heated to reflux. During the heating reflux process, the reaction mixture was dissolved first, and then a large amount of orange solids precipitated. The reaction mixture was cooled to the room temperature and was filtered into the crude product. The crude product was re-crystallized from ethanol to obtain an orange solid with a yield of 85%.

^1^H NMR (400MHz, DMSO-d6) δ 9.12 (s, 1H), 8.61 (d, *J* = 8.8 Hz, 1H), 8.41 (d, *J* = 8.8 Hz, 1H), 8.29 (d, *J* = 8.6 Hz, 1H),7.63 (t, *J* = 7.4 Hz, 1H), 7.24 (d, *J* = 8.6 Hz, 1H), 4.67 (s, 2H), 4.01 (t, *J* = 7.4 Hz, 2H), 1.58 (m, 2H), 1.33 (m, 2H), 0.92 (t, *J* = 7.4 Hz, 3H). ^13^C NMR (100 MHz, DMSO-d6) δ 164.2, 163.4, 153.6, 134.6, 131.0, 129.7, 128.7, 124.6, 122.2, 118.9, 107.8, 104.4, 40.6, 30.3, 20.3, 14.2. HR-MS calculated for C16H17N3O2 [M+H]^+^ m/z 284.1399, found 284.1395.

1.4. Cellular Viscosity Imaging

For the viscosity-responsive confocal imaging, the Hela cells were first cultured with normal, monensin, and nystatin (both at 20 μM) for 50 min. After washing with PBS three times, the freshly prepared stock solutions of **HA-Na** (10 μM in DMEM) were added to the above cell plates, which were further cultured at 37 °C for 30 min. The cells were excited at 488 nm and emissions were collected at 500–600 nm with a 63× oil-immersion objective lens.

1.5. Cellular Autophagy Monitoring

For monitoring the autophagy process through the lysosomal viscosity changes. Hela cells were incubated with HA-Na (10 μM) at 37 °C for 30 min and then were cultured in HBSS, normal medium, or HBSS with the addition of 3-methyladenine (3-MA, 100 μM) for 90 min, respectively. The cells were excited at 488 nm and emissions were collected at 500–600 nm with a 63× oil-immersion objective lens.

**2. Computational methods**

All molecular structures in the ground and excited states were optimized at M06-2X/TZVP level. The M06-2X functional has been wieldy applied to optimize the structures of organic molecules and calculate the excited state properties of dyes ^1, 2^. The solvent effect was (water) included via the SMD model in all calculations ^3^. The frequency values were analyzed to confirm the minimum point without imaginary frequency values. All calculations were carried out using the Gaussian 16 code ^4^. VMD 1.9.3 and Multiwfn 3.7 ^5^ were used to visualize the distributions of frontier molecular orbit.


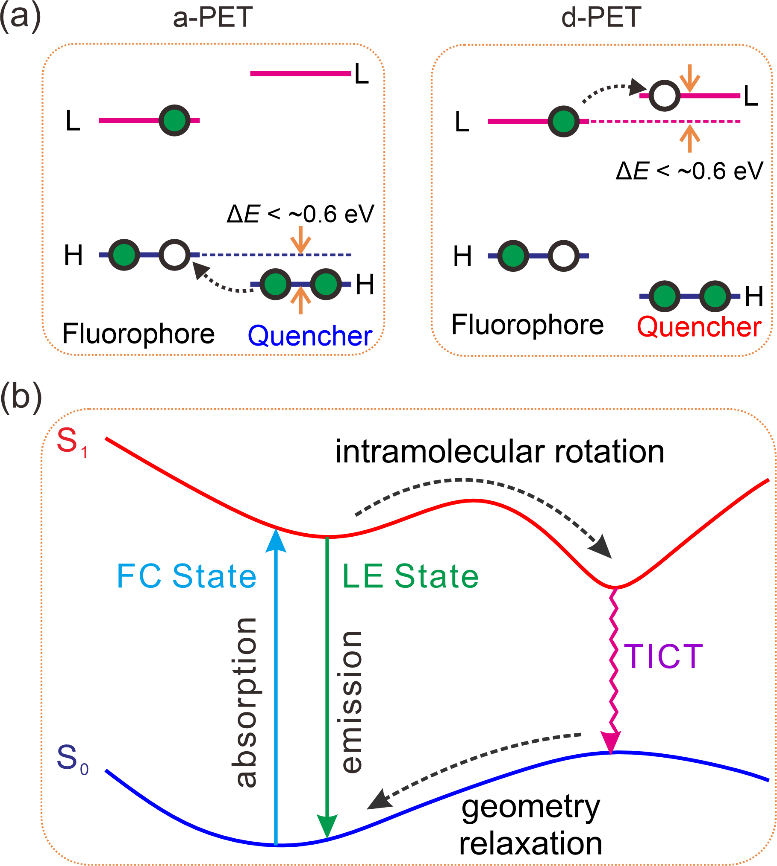


**Figure S1.** (a) orbital model of the PET mechanism, (b) diagram of the ground state and excited state potential energy surface of TICT mechanism.


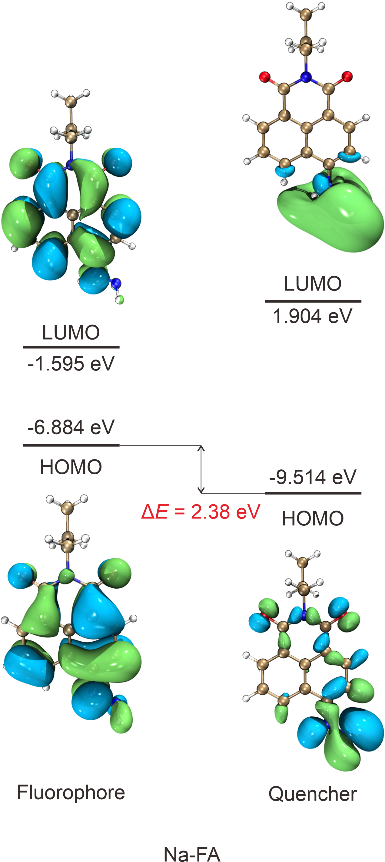


**Figure S2.** Calculated distributions of HOMO and LUMO in fluorophore and quencher for Na-FA and the energy gaps between HOMOs at M062X/TZVP level.


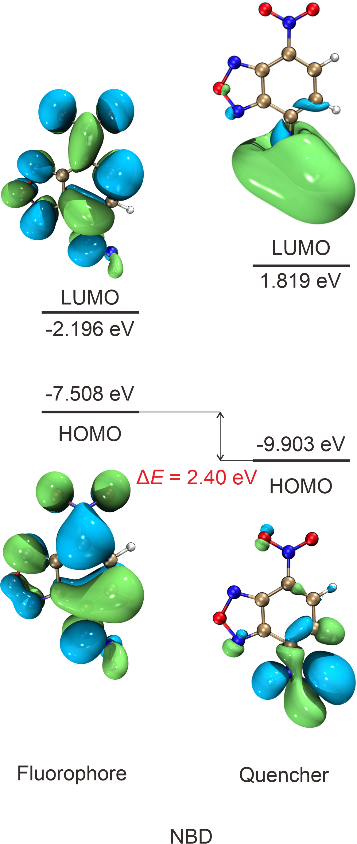


**Figure S3.** Calculated distributions of HOMO and LUMO in fluorophore and quencher for NBD and the energy gaps between HOMOs at M062X/TZVP level.


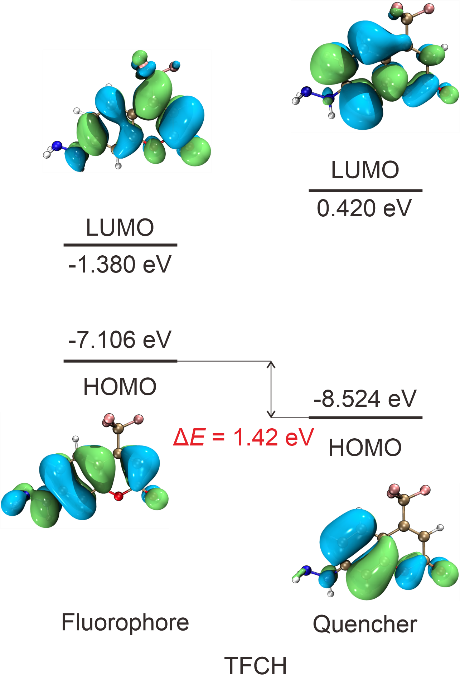


**Figure S4.** Calculated distributions of HOMO and LUMO in fluorophore and quencher for TFCH and the energy gaps between HOMOs at M062X/TZVP level.


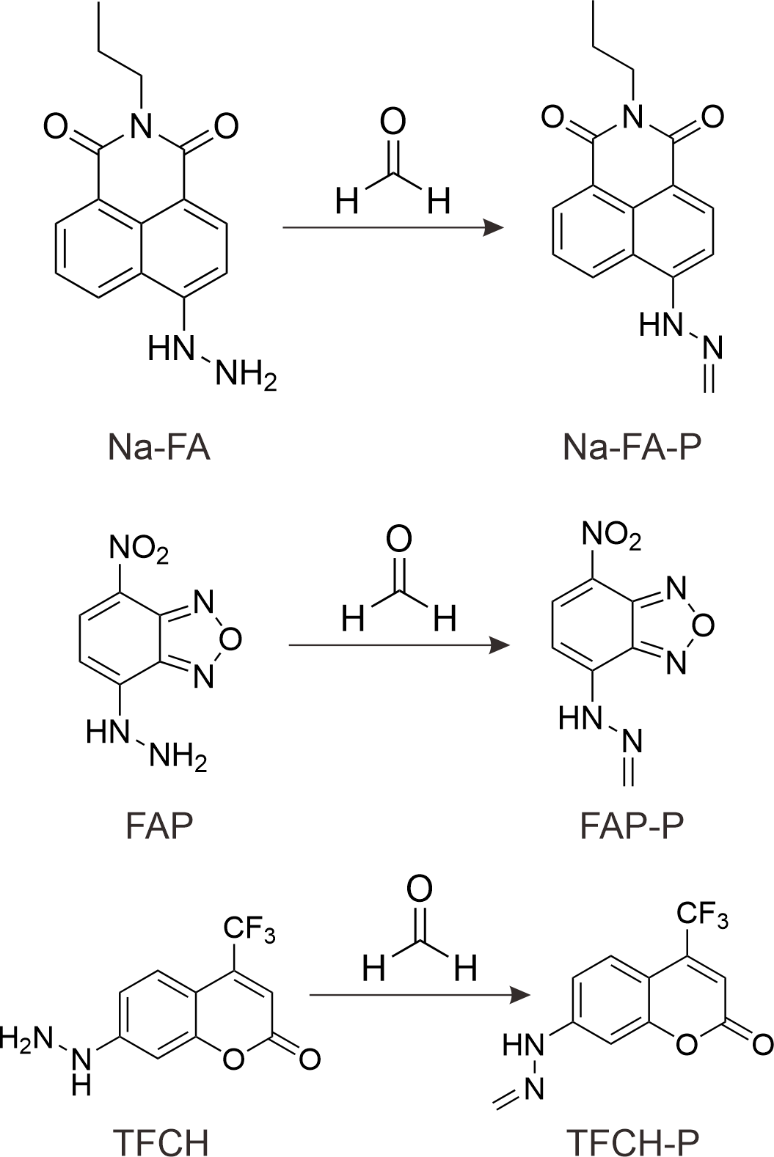


**Figure S5.** The reaction products of Na-FA, FAP, TFCH with formaldehyde.


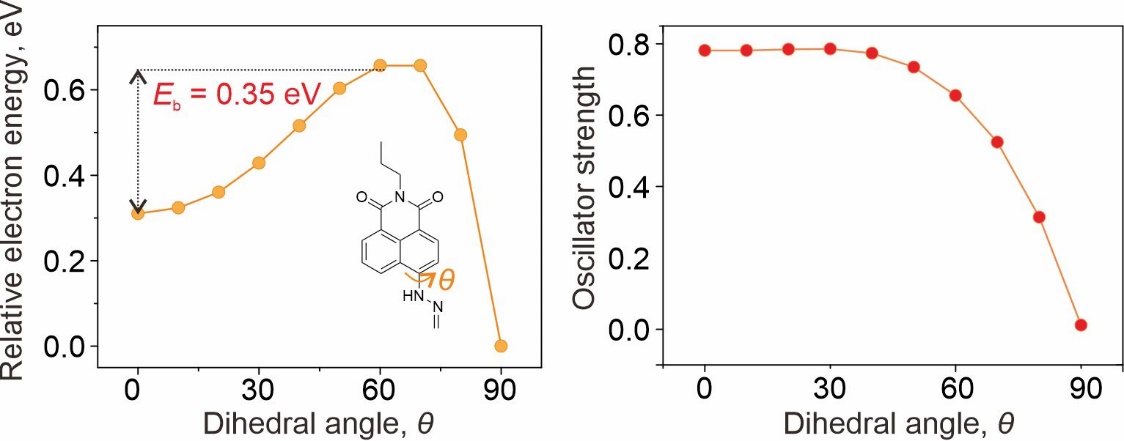


**Figure S6.** The excited state PESs of Na-FA-P as a function of the rotation of the methylenehydrazine group in water solution and the corresponding oscillator strength.


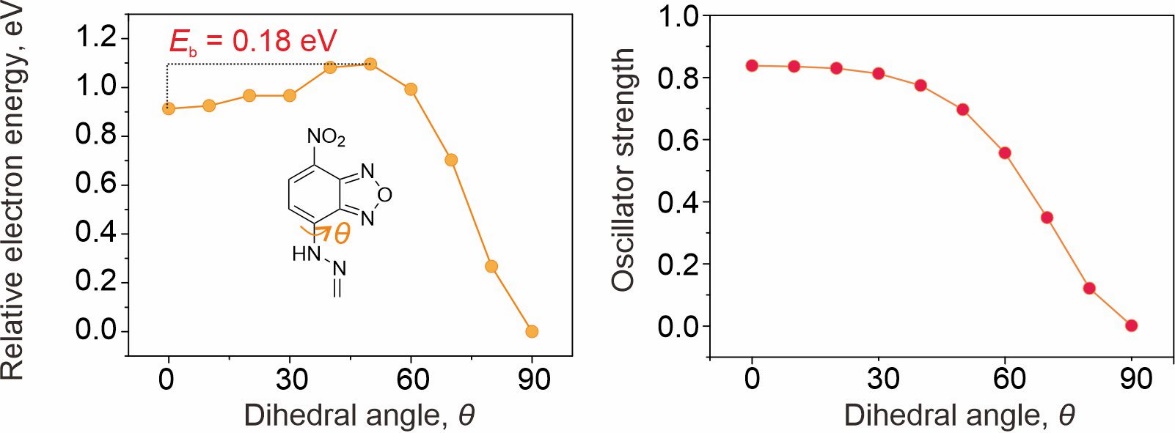


**Figure S7.** The excited state PESs of FAP-P as a function of the rotation of the methylenehydrazine group in water solution and the corresponding oscillator strength.


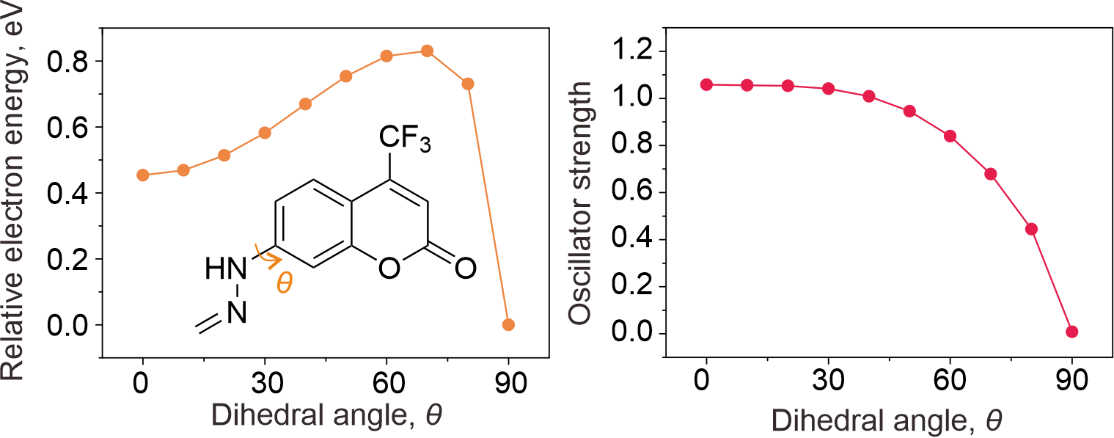


**Figure S8.** The excited state PESs of TFCH-P as a function of the rotation of the methylenehydrazine group in water solution and the corresponding oscillator strength.


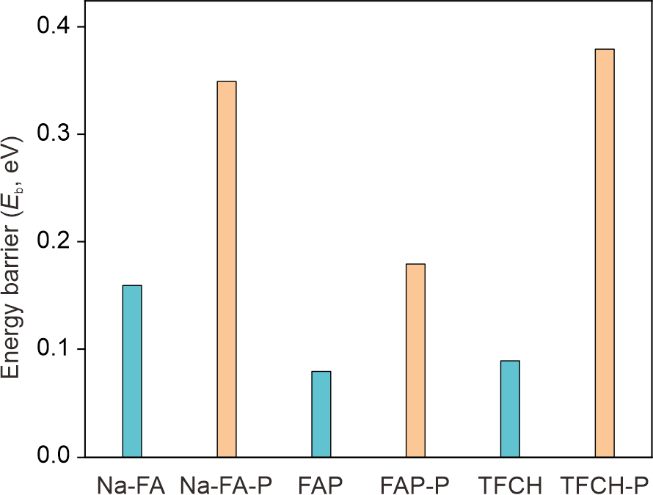


**Figure S9.** The energy barrier values of formation of TICT state in water.


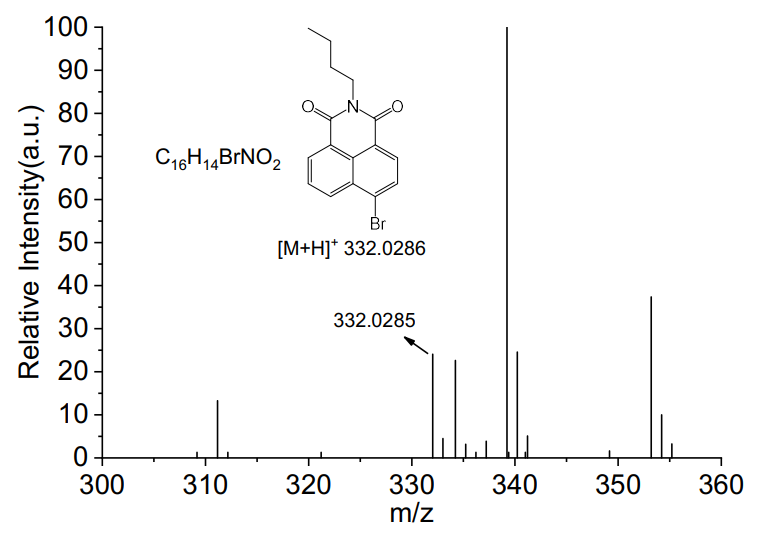


**Figure S10.** HR-MS spectrum of the compound **2**.


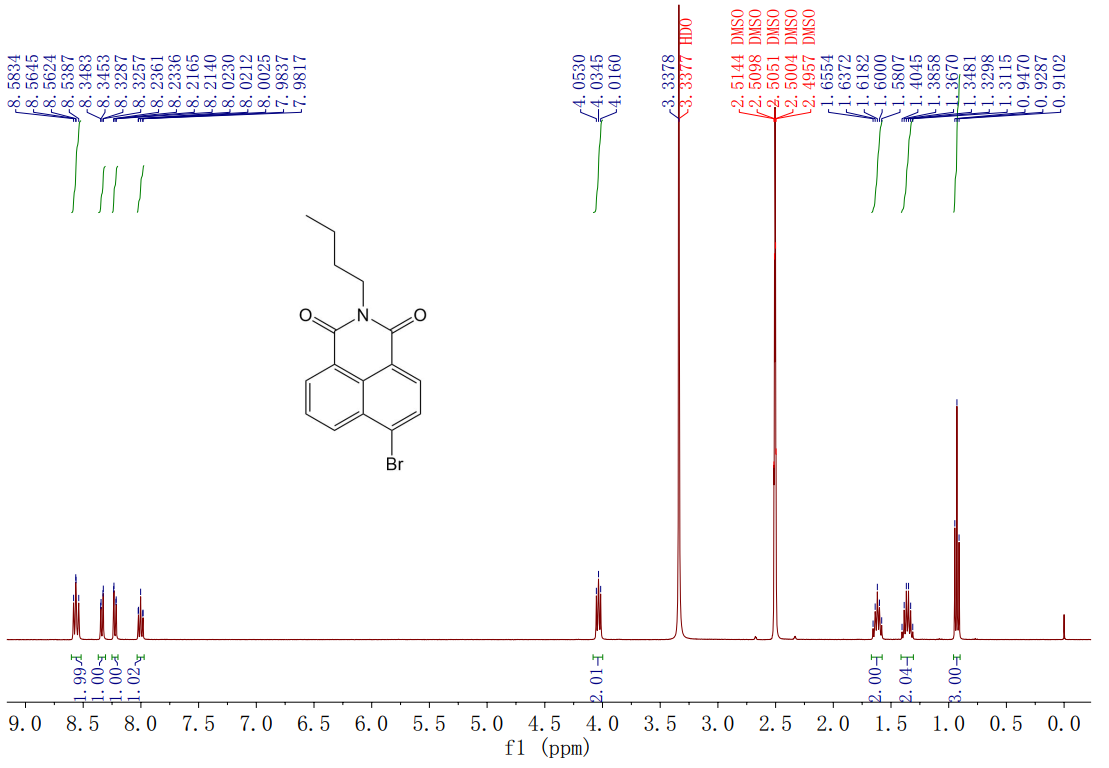


**Figure S11.** ^1^H NMR spectrum of the compound **2**.


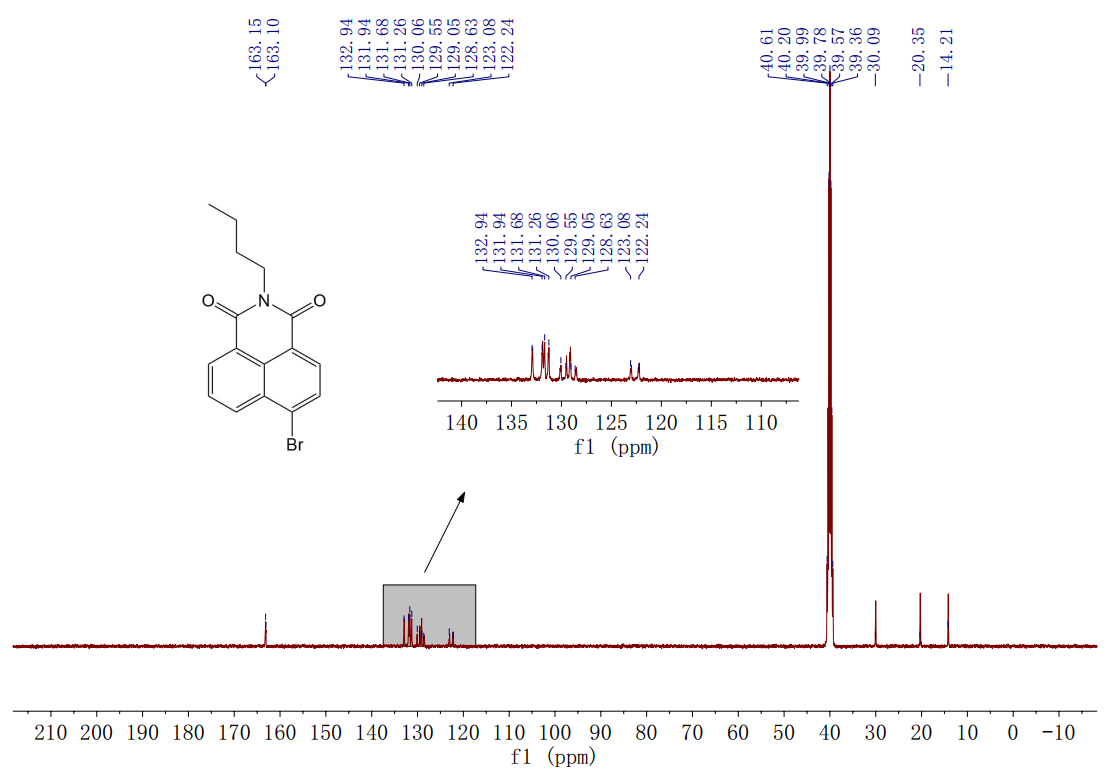


**Figure S12.** ^13^C NMR spectrum of the compound **2**.


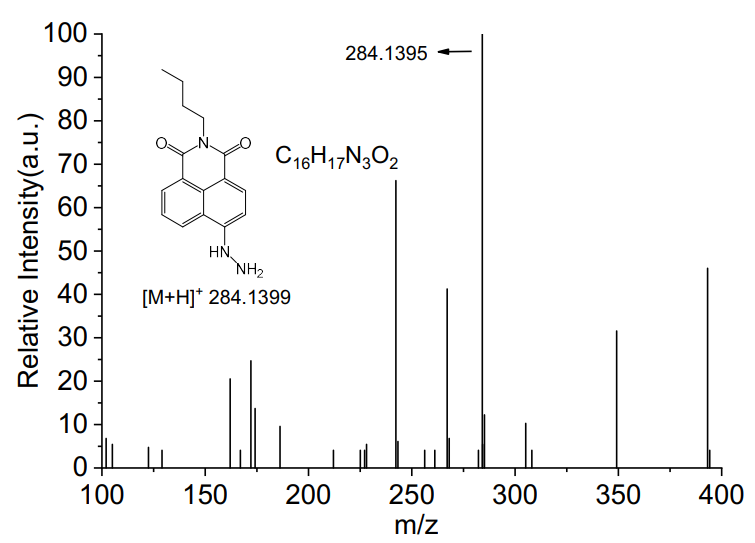


**Figure S13.** HR-MS spectrum of the compound **HA-Na**.


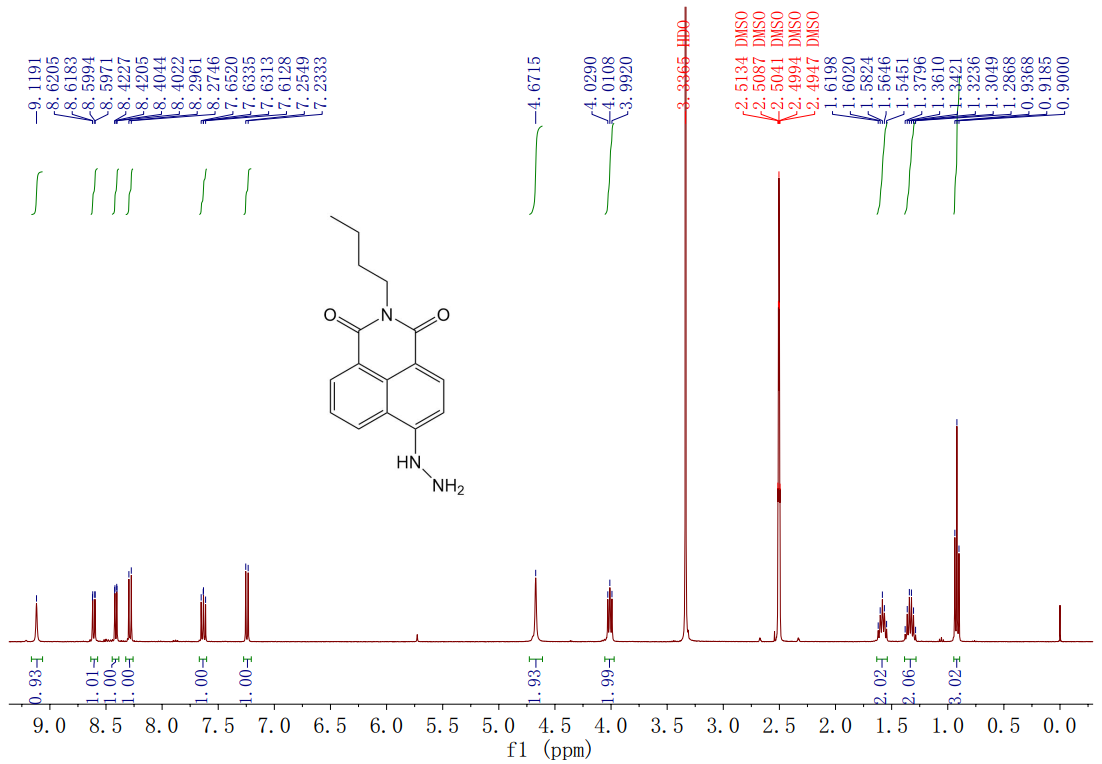


**Figure S14.** ^1^H NMR spectrum of the compound **HA-Na**


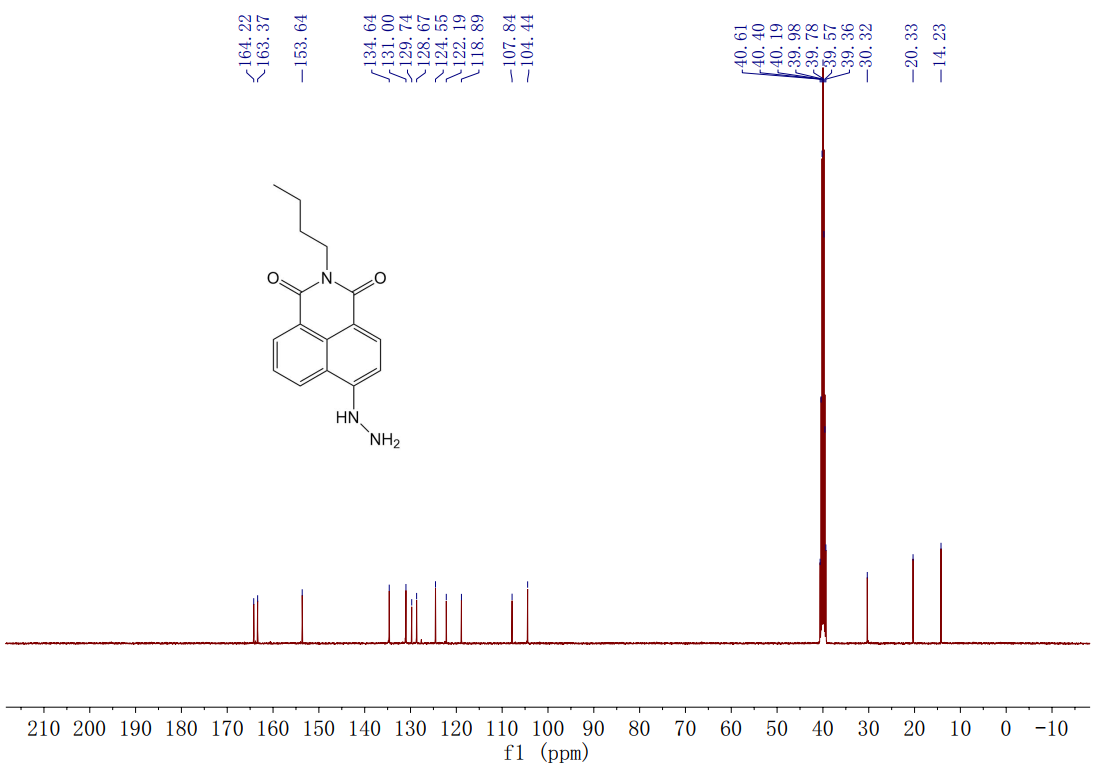


**Figure S15.** ^13^C NMR spectrum of the compound **HA-Na**.


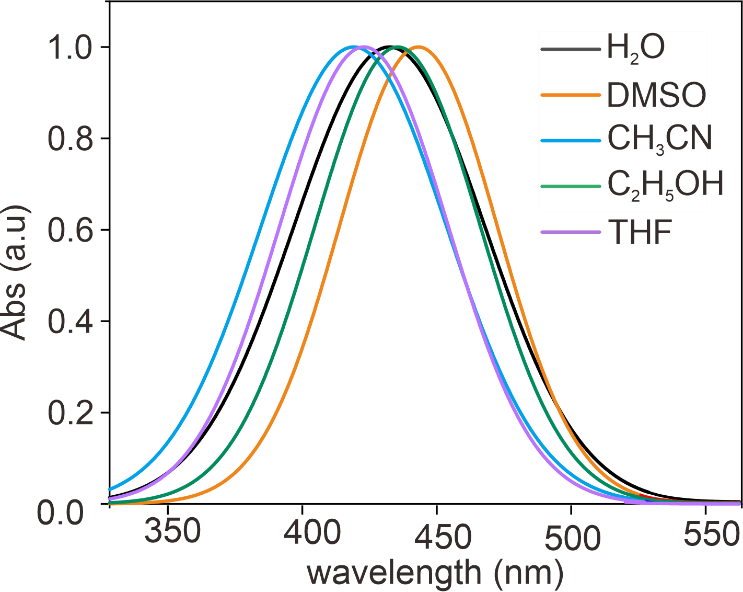


**Figure S16.** The absorption spectra of HA-Na in water, DMSO, acetonitrile, ethanol, and tetrahydrofuran.


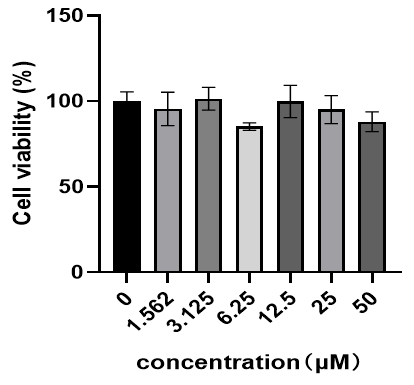


**Figure S17**. MTT assay of Hela cells with different concentration of 1 (0-50 uM)

for 24 h.


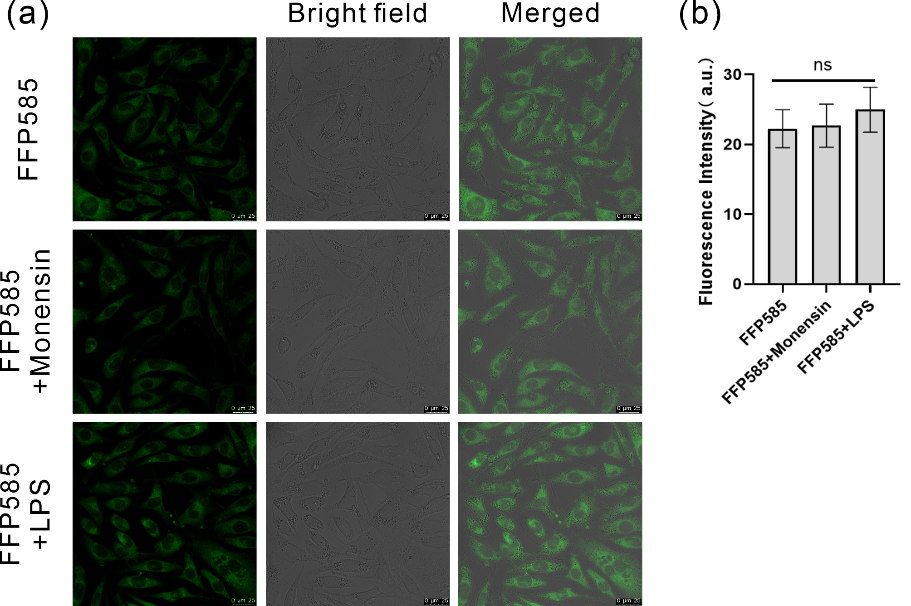


**Figure S18** (a) Confocal fluorescence images of FFP585 (5*μ*M) in Hela cells, which were pre-treated with normal, monensin(20*μ*M), and LPS(100ng/ml) for 40 min and then treated with FFP585 (5*μ*M) for another 40 min. (b)the relative fluorescence intensity with normal, monensin, and LPS. (λ_ex_ = 514 nm, collected 534-700 nm. Scale bars are 25 μm).


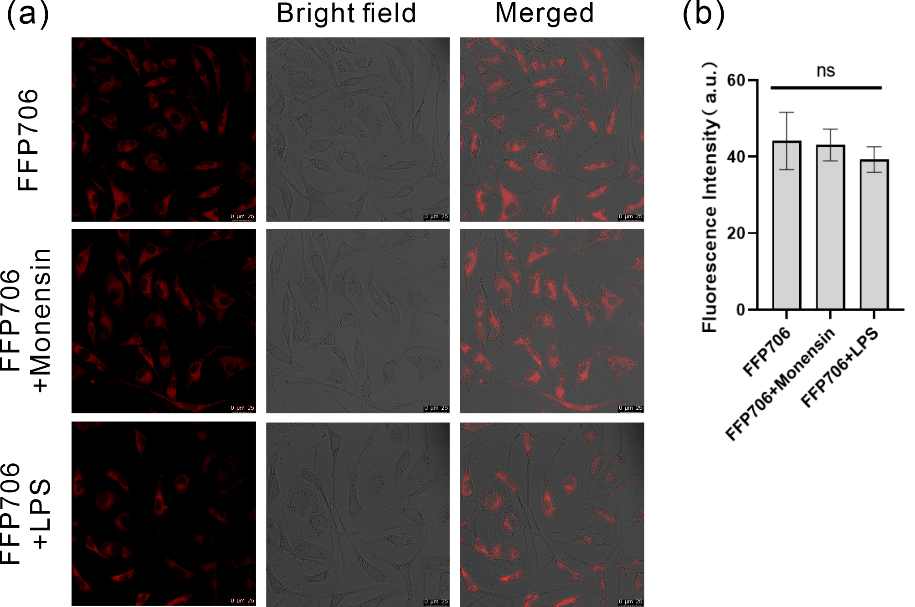


**Figure S19** (a) Confocal fluorescence images of FFP706 (5*μ*M) in Hela cells, which were pre-treated with normal, monensin(20*μ*M), and LPS(100ng/ml) for 40 min and then treated with FFP706 (5*μ*M) for another 40 min. (b)the relative fluorescence intensity with normal, monensin, and LPS. (λ_ex_ = 633 nm, collected 633-750 nm. Scale bars are 25 μm).

**References**

1. W. Chi, Q. Qi, R. Lee, Z. Xu and X. Liu, *The Journal of Physical Chemistry C*, 2020, **124**, 3793-3801.

2. W. Chi, Q. Qiao, C. Wang, J. Zheng, W. Zhou, N. Xu, X. Wu, X. Jiang, D. Tan, Z. Xu and X. Liu, *Angewandte Chemie International Edition*, 2020, **59**, 20215-20223.

3. A. V. Marenich, C. J. Cramer and D. G. Truhlar, *The Journal of Physical Chemistry B*, 2009, **113**, 6378-6396.

4. M. e. Frisch, G. Trucks, H. Schlegel, G. Scuseria, M. Robb, J. Cheeseman, G. Scalmani, V. Barone, G. Petersson and H. Nakatsuji, *Journal*, 2016.

5. T. Lu and F. Chen, *Journal of Computational Chemistry*, 2012, **33**, 580-592.
